# Effect of Panel Moisture Content on Internal Bond Strength and Thickness Swelling of Medium Density Fiberboard

**DOI:** 10.3390/polym13010114

**Published:** 2020-12-30

**Authors:** Roberto Magalhães, Beatriz Nogueira, Samaritana Costa, Nádia Paiva, João M. Ferra, Fernão D. Magalhães, Jorge Martins, Luisa H. Carvalho

**Affiliations:** 1 EuroResinas—Indústrias Químicas, 7520-195 Sines, Portugal; Roberto.Magalhaes@sonaearauco.com (R.M.); Beatriz.Nogueira@sonaearauco.com (B.N.); Samaritana.Costa@sonaearauco.com (S.C.); nadia.paiva@sonaearauco.com (N.P.); joao.ferra@sonaearauco.com (J.M.F.); 2 LEPABE—Laboratory for Process Engineering, Environment, Biotechnology and Energy, Faculty of Engineering, University of Porto, 4200-465 Porto, Portugal; fdmagalh@fe.up.pt (F.D.M.); jmmartins@estgv.ipv.pt (J.M.); 3 DEMad—Departamento da Engenharia de Madeiras IPV, 3504-510 Viseu, Portugal

**Keywords:** wood-based panels, commercial MDF, storage conditions, physico-mechanical properties

## Abstract

Wood-based products usually have serious limitations concerning contact with water, both because wood is a hygroscopic material and because the commonly used binder has low moisture resistance. This paper studies the effect of panel moisture content (MC) on the physico-mechanical properties of medium density fiberboards (MDF). Several commercial MDF boards produced in Europe were stored at room temperature and relative humidity (RH) for 9 weeks (approx. range 15–20 °C and 50–85% RH). Every week, a strip of each MDF board was cut out, divided into 5 × 5 cm test pieces and its internal bond strength (IB) was measured. A strong influence of MDF moisture content on internal bond strength was observed and therefore IB test pieces were stored in a climatic chamber (either at 20 °C, 55% RH and at 20 °C, 70% RH). A decreasing linear relation was established between IB and MC. It was found that this effect is reversible: after drying, internal bond strength rises again (following a slight hysteresis). This work reinforces the importance of conditioned storage before board properties analysis, as described in European Standard EN 319.

## 1. Introduction

Dry process fiberboard, commercially known as Medium Density Fiberboard (MDF), is a wood-based panel (WBP) that is composed of wood fibers bonded with a thermoset resin and an additive, such as wax, in minor amount. The board is produced under heat and pressure. The resins in use are typically formaldehyde-based: phenol-formaldehyde (PF), urea-formaldehyde (UF) and melamine-urea-formaldehyde (MUF) [1]. UF resins are the most common, due to their high reactivity, low cost and excellent adhesion to wood [2].

MDF is widely used in the building industry as an eco-friendly alternative to solid wood due to a good combination of mechanical and thermal properties and competitive price. The hot-pressing operation is the final stage in the MDF manufacture where the mattress of fibers is compressed and heated to promote the cure of the resin. The press cycle has though a major effect on the balance of properties of the resulting panel. The rheological behavior, heat and mass transfer and resin cure are complex phenomena that are dependent on temperature, moisture content, vapor pressure and density distribution [3]. Upon leaving the press, most boards are cooled, generally in a star cooler, to help balance moisture content distribution and stresses. The temperature, moisture content and vapor pressure changes dynamically during hot pressing, but it continues during stacking (after cooling) and these conditions will also affect the final board properties [4].

During the pressing operation, an appropriate amount of water is necessary to transfer heat from the outer layers to the center layer of mats. The amount of moisture in wood prior to pressing will greatly influence wetting, penetration and cure of adhesives. If moisture content is too low during pressing, wood will absorb water from the adhesive so quickly that its flow and penetration into the wood is inhibited. When wood contains excess amounts of moisture, this leads to excessive adhesive mobility, followed by squeeze-out when pressure is applied. Control of moisture content is particularly critical in the hot-pressing operation [5].

Raw materials as fiber’s characteristics and the resin’s chemical bonds will also affect panel properties, mostly the mechanical strength. Higher strength and dimensional stability are normally expected with increasing resin level. Maloney [6] described that longer fibers originate an increased network system and result in improved mechanical properties of wood-based panels. Resin type influences the thickness swelling and spring-back of the board, even in high density layers, the fiber-resin bond being the limiting factor [7]. Resin consumption is an important parameter that has to be optimized, because of its relevant impact on the final cost of the product.

The resin is the main source of formaldehyde emission of WBPs. This compound is classified as a possible health hazard following a World Health Organization (WHO) recommendation published in 2006 [8]. Since then, several efforts have been made in order to reduce formaldehyde from WBPs [9]. The emissions are mainly due to the presence of unreacted free formaldehyde remaining after curing, as well as from hydrolysis of methylene-ether bridges [10]. Lowering the free formaldehyde content typically leads to lower resin reactivity, which may causes a reduction of the mechanical properties of the boards [11,12]. Some regulations limiting the emission of formaldehyde have been defined, as the European Standard EN 622-1 [13] (E1 or E2 board) and the California Air Resources Board phase II [14] (CARB II board) emission standards. Structural and non-structural wood-based composites such as MDF, oriented strand board (OSB), plywood, etc., are now used for both interior and exterior applications. Their use, however, is often limited since the environmental conditions, in certain application classes, can be so adverse that their performance is negatively affected [15]. Water sorption and desorption cycles generate swelling and shrinkage. This affinity of WBPs towards water is caused by hydroxyl groups in the wood cell walls [16].

When in contact with surrounding air with a relative humidity of nearly 100% or in the presence of liquid water, wood cell walls get saturated, though attaining the fiber saturation point and hence total swell. This is the critical point at which wood will begin to shrink if the relative humidity of the surrounding air decreases. If wood dries below the fiber saturation point and then regains moisture, it will swell again [17]. This behaviour towards water is strongly influenced by adhesives, board density and production technology (drying, defibration, press cycle temperature and pressure, stacking, etc.). Repeated changes in ambient conditions increase the probability of wood developing defects in its structure, affecting also the final product [18].

Water-induced swelling is largely reversible for solid wood, but this is not the case for particleboards, fiberboards and compressed wood. The equilibrium moisture content (EMC) of WBPs—the condition where the material stabilizes its weight under constant RH—is lower than for solid wood of the same species and under the same conditions, due to the influence of particle drying (thermal modification), defibration (physical modification), hot-pressing (thermal or hydrothermal treatment, densification), and the presence of resin (chemical modification). For instance, EMC of MDF is correlated to the defibration process—the thickness swelling of MDF decreases when high pressures and long defibration durations are employed [19].

The amount of water held by cellulosic materials depends both on the equilibrium relative vapor pressure and the direction from which equilibrium is approached (adsorption or desorption). This difference is generally referred to as sorption hysteresis. Hysteresis is normally related to capillary condensation in micropores and expansion of the porous structure during adsorption, and to reduction of the availability of sorption sites during drying, as OH groups bond with each other as they draw closer due to shrinkage [20,21].

MC is indirectly related to RH and reaching EMC may take a very long time depending on the rate of diffusion of water vapor into the board. Diffusion rates are controlled primarily by panel density. In most cases, all board properties decrease as the temperature and moisture levels increase. Well-made boards with any of the commonly used adhesives will retain their long term stability if the moisture content of the wood does not exceed approximately 15% and if the temperature remains within the range of human comfort [22]. Consequently, MC is one of the most important variables affecting its physical and mechanical properties.

In this study, commercial MDF samples manufactured in Europe were characterized in order to evaluate the influence of panel moisture content on its physico-mechanical properties, namely internal bond strength and thickness swelling.

## 2. Materials and Methods

### 2.1. Test Samples

Three commercial samples of general purpose MDF for dry conditions, with different formaldehyde emission classification (samples A, B and C, emission class CARB II or E1, as described in Table 1), manufactured in Europe, were tested in this work. Formaldehyde based amino resins were used for all boards. All the samples were bought in a trader warehouse. CARB (California Air Resources Board) governs air quality, namely formaldehyde emissions. Phase II of CARB’s Airborne Toxic Control Measure (ATCM) went into effect in California in 2010. The limits of formaldehyde emission for MDF under CARB II (Phase II) rule is 0.11 ppm. The European class E1, as defined in EN 13986 + A1 considers a limit of 0.124 mg/m^3^ air (equivalent to 0.1 ppm) for formaldehyde emission determined using the chamber method EN 717-1.

A total of six MDF boards were cut, two from each sample, each with approx. 50 cm × 50 cm wide.

The mean density and thickness of the test pieces (measured just before the IB tests) is shown in Table 1. These properties remained nearly constant for all samples throughout the study period.

The samples were stored at the laboratory and were subjected to the natural daily changes of the laboratory temperature and relative humidity over 9 weeks for samples A and B, 6 weeks for sample C (approx. range 15–20 °C and 50–85% RH).

Samples A and B were tested first. Every week a strip of each sample was cut out and divided into 5 cm × 5 cm test pieces for further characterization. A strong influence of MDF moisture content on internal bond strength was observed and thereby test pieces of all samples were also stored in climatic chambers (Weiss Tecknik WK3-340/40) under two different conditions: 20 °C, 55% RH and 20 °C, 70% RH.

### 2.2. Determination of Physico-Mechanical Properties

Test pieces with 5 cm × 5 cm × thickness from each sample were used for each test. The samples conditioning requirement established in the European standards (20 °C and 65% RH) was not applied since the purpose of the study was to study the effect of moisture content.

Thickness Swelling (TS) was evaluated according to the test method of EN 317 [23] after 24 h of water immersion.

Internal Bond Strength (IB) tests were carried out on IMAL board property tester IB700 according to EN 319 [24]. The load was continuously applied to the test pieces at a constant rate of 1 mm/min assuring that the maximum load was reached within (60 ± 30) s.

Moisture content (MC) was evaluated according to EN 322 [25] and density was evaluated according to EN 323 [26].

The reported results are average values of at least four replicates for the IB at room conditions and at least two replicates for all the other measured properties.

## 3. Results and Discussion

In parallel with the physico-mechanical properties determined at room conditions, in the range 15–20 °C and 50–85% RH during the test period, test pieces of samples A and B in the last two weeks (and sample C over the first 6 weeks) were stored in climatic chambers under two different conditions: 20 °C, 55% RH and 20 °C, 70% RH.

Moisture content, internal bond, thickness swelling and average standard deviation results are shown below on Table 2 and Table 3 for further discussion on next sections.

The climatic chamber storage, for each sample and RH condition, was performed for at least 4 days for samples A and B. Sample C was stored until reaching the stability criteria: constant mass is considered to be reached when the results of two successive weighing operations, carried out at an interval of 24 h, do not differ by more than 0.1% of the mass of each test piece (EN 321).

### 3.1. Thickness Swelling

**Figure 1:**
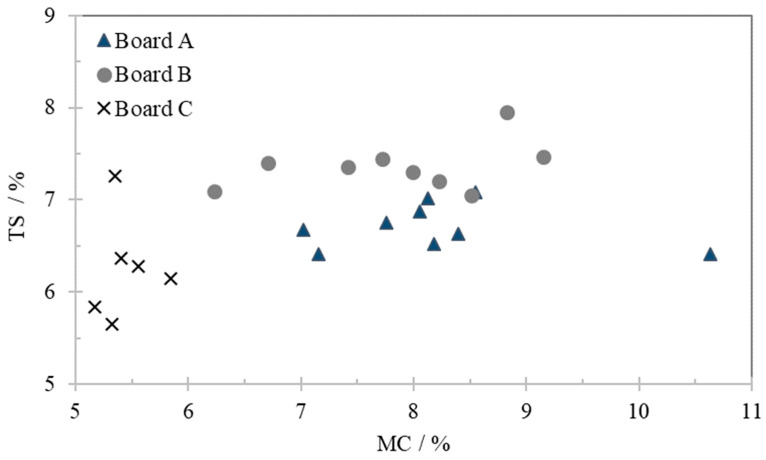
Thickness swelling dependence on moisture content for samples stored at room temperature and relative humidity.

Figure 1 shows all the TS and MC values for the three samples during the test period (9 weeks for boards A and B and 6 weeks for board C—one set of data per week). The TS value of samples A and B did not show any significant variation with MC. Sample C presents a higher variability, however the highest and lowest values occurred within the first 2 weeks (see Table 2 and Table 3), so there is no chronological trend in the values obtained. In this measurement range the experimental error is significant since the thickness is measured only at the central point of the test piece, while the thickness might not be uniform along the test piece by the end of the TS test.

It is known that relative humidity (RH) influences the thickness swelling. Wood composites are viscoelastic and porous materials and the thickness swelling occurs primarily because of the swelling of the wood elements. Thickness swelling of wood composites can also have a negative effect on other mechanical properties of the board.

Some studies [27,28] report the poor resin resistance against hydrolysis, specially for UF resins at high relative humidity or temperature, due to the weak bonding between the nitrogen of the urea and the carbon of the methylene bridge. Resin hydrolysis also leads to an irreversible loss of bonding strength and there is no such effect in this study.

Halvarsson et al. [29] tested several samples with different densities (all above 750 kg·m^−3^) and for the same resin blend no evident correlation between TS and density was found. Nonetheless higher resin content or higher melamine content of MUF resins had a greater impact, decreasing the TS.

In this study no relationship was found between the TS and MC. TS values for all boards were well within the limits of European standard EN 622–5 (requirements for dry process boards) wherein the specification for use in dry conditions is TS lower than 12%.

### 3.2. Internal Bond Strength

The average IB values for all boards met the minimum specification requirement (0.55 N·mm^−2^) of EN 622–5 for general purpose boards for use in dry conditions at the beginning of the test (1st week).

**Figure 2:**
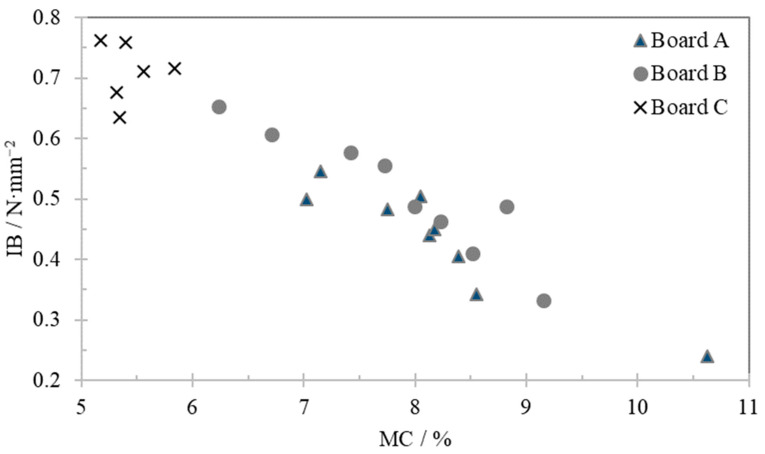
Internal bond dependence on moisture content for samples stored at room temperature and relative humidity.

Figure 2 shows the relation between IB and MC for the three boards stored at room conditions during the test period (one set of data per week). IB values tend to decrease over time with the increase of MC on samples A and B and remained stable for sample C (values on Table 2 and Table 3).

A decreasing linear relation can be observed between IB and MC. Sample C showed a significantly higher stability (and density—see Table 2) than samples A and B. In the case of board C, the water sorption is lower than for boards A and B. Since no information was available about the production parameters for board C (like resin or wax emulsion contents), it is impossible to explain the reason why board C displays less water adsorption than boards A and B under the same conditions.

Boards A and B have different formaldehyde emission classification, CARB II and E1, respectively which probably means different levels of formaldehyde emissions and though different resin type and additives formulation, as formaldehyde scavengers, but these differences seems not to have impact on IB value. On the other hand, boards C presents completely different results, which indicates that sample’s density has an impact on IB values.

Dunky and Pizzi [27] described that the bond strength increases with the board density in the range of about 700 to 800 kg·m^−3^. The performance of the boards is also strongly influenced by the wood quality (biological degradation, use of burned wood, etc.) and species used. The decrease of the mechanical properties with increase of moisture content in the MDF boards can be explained by the lower fiber stiffness. Maloney [6] observed a negative correlation between the moisture content and IB values of the boards.

IB strength results from the resistance of resin-resin and resin-wood fiber bonds which are affected by resin cure degree, adhesion on the interface and cohesion inside the glueline. Being the result of a tensile test, the strength of each bond depends on local elongation which depends on the local stiffness of the materials. Wood fibers in MDF are organized in a complex 3D structure. This structure is a direct consequence of the forming and pressing operations, but also of the fiber morphology, deformation, slipping, intersection, and the settlement of resin bridges [30]. As a consequence, the local strain distribution is wide. Wood fibers properties have an anisotropic behaviour. When the fibers moisture content increase, there is swelling and stiffness reduction of fibers, which are different in each fiber directions (axial and radial). These results in an even wider distribution of local strains, and consequently a lower total strength (IB).

**Figure 3:**
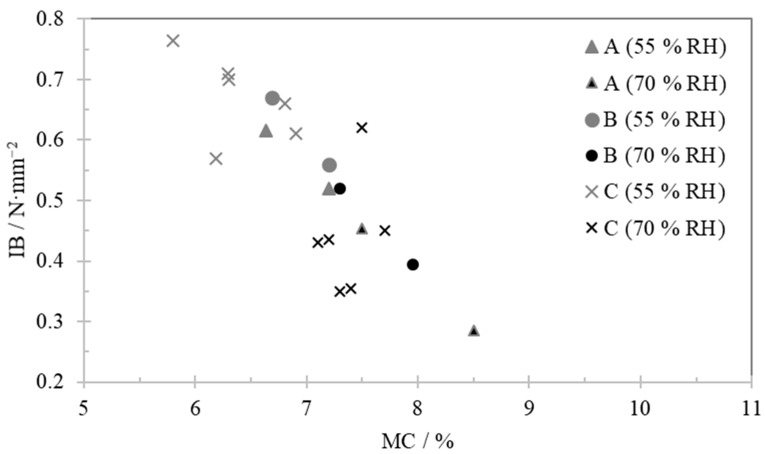
Internal bond dependence on moisture content for samples stored in the climatic chamber.

Figure 3 shows the relation between IB and MC for the samples that were conditioned in a climatic chamber before testing. As expected, the samples conditioned at 55% RH display lower MC than the ones conditioned at 70%.

A linear relation can be observed between these two properties, however the equilibrium moisture content (EMC) was not fully achieved as, for the same sample (board type), it would be expected to have the same MC values every week, under the same climate conditions.

By comparison of Figure 2 and Figure 3, the same trend of MC dependency is observed. All samples stored at 55% RH performed similarly to samples tested in the first week.

**Figure 4:**
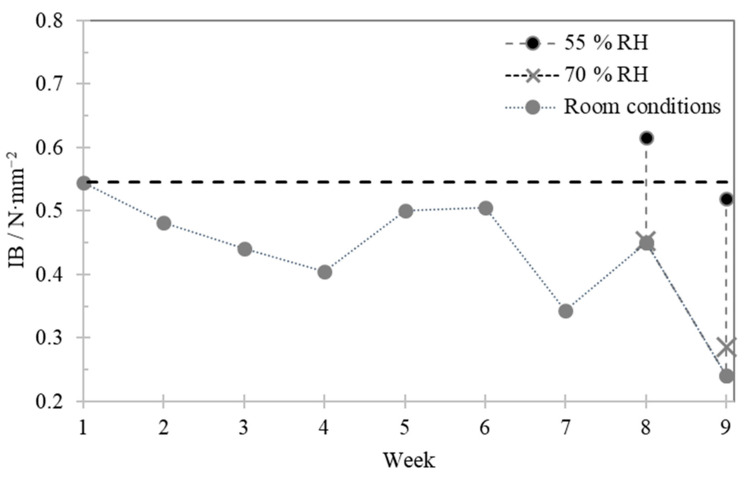
Internal bond history for sample A during the 9 week test period.

Figure 4 shows the internal bond history for sample A. At room conditions, IB values decreased over time and after storage at 55% RH, in the last two weeks, similar IB values to those of the 1st week at room conditions were achieved.

The storage relative humidity on samples A and B proved to have much impact in their mechanical properties. The results reinforce the importance of board producers to adequately climate their warehouses.

## 4. Conclusions

This study describes the impact of indoor climate (of particular interest in post-production storage) on the adsorption and desorption of moisture in MDF boards. The results showed that the largest MC fluctuation occurred on samples A and B (which have lower densities). Sample C showed a significantly higher stability concerning the IB results, even though it follows the overall trend of MC dependency. These results reinforce the importance of conditioning before testing MDF boards, as described in European Standard EN 319 (20 °C, 65% RH).

The main conclusion of this study is that the IB decreases with the increase of MC and this effect is reversible by sample drying, with a slight hysteresis. This reversible behavior seems to indicate that the reduction of IB when MC increases is not related to resin hydrolysis but rather probably to lower fiber stiffness. As we described before the board density had a greater impact than the formaldehyde emission level (owing to different resin type or content on the tested samples) on the dimensional stability of the MDF boards. The variation of MC was slightly for sample C, when stored at room temperature and RH.

The TS shows a poor relationship with MC and this is likely because these boards already have very low swelling values. Because water sorption process is very slow, the moisture history of MDF in dynamic environments is complex and the improvement of the board’s mechanical stability implies further studies.

## Figures and Tables

**Table 1:** Density and thickness of MDF samples.

Sample	Density (kg·m^−3^)	Thickness (mm)
A—CARB II board	689 ± 3	19.24 ± 0.16
B—E1 board	692 ± 4	19.48 ± 0.15
C—CARB II board	763 ± 6	19.13 ± 0.03

**Table 2:** Results for MDF board stored at room temperature and RH.

	Stored at Room Temperature and RH (Non-Fixed Conditions)								
	A			B			C		
Week	MC	IB	TS	MC	IB	TS	MC	IB	TS
1	7.2	0.55	6.4	6.2	0.65	7.1	5.3	0.68	5.7
2	7.8	0.48	6.8	7.4	0.58	7.4	5.3	0.64	7.3
3	8.1	0.44	7.0	8.0	0.49	7.3	5.4	0.76	6.4
4	8.4	0.41	6.6	8.5	0.41	7.1	5.8	0.72	6.1
5	7.0	0.50	6.7	6.7	0.61	7.4	5.6	0.71	6.3
6	8.1	0.51	6.9	7.7	0.56	7.4	5.2	0.76	5.8
7	8.6	0.34	7.1	8.8	0.49	7.9	---	---	---
8	8.2	0.45	6.5	8.2	0.46	7.2	---	---	---
9	10.6	0.24	6.4	9.2	0.33	7.5	---	---	---
Stdev	±0.2	±0.04	±0.1	±0.3	±0.05	±0.3	±0.1	±0.05	±0.2

RH, relative humidity; MC, moisture content (%); IB, internal bond (N·mm^−2^); TS, thickness swelling (%).

**Table 3:** Results for MDF boards stored in climatic chamber.

	Stored at 20 °C, 55% RH						Stored at 20 °C, 70% RH					
	A		B		C		A		B		C	
Week	MC	IB	MC	IB	MC	IB	MC	IB	MC	IB	MC	IB
1	---	---	---	---	6.3	0.70	---	---	---	---	7.3	0.35
2	---	---	---	---	5.8	0.77	---	---	---	---	7.1	0.43
3	---	---	---	---	6.3	0.71	---	---	---	---	7.2	0.44
4	---	---	---	---	6.2	0.57	---	---	---	---	7.7	0.45
5	---	---	---	---	6.9	0.61	---	---	---	---	7.4	0.36
6	---	---	---	---	6.8	0.66	---	---	---	---	7.5	0.62
7	---	---	---	---	---	---	---	---	---	---	---	---
8	6.7	0.62	6.7	0.67	---	---	7.5	0.45	7.3	0.52	---	---
9	7.2	0.52	7.2	0.56	---	---	8.5	0.29	8.0	0.40	---	---
Stdev	±0.04	±0.02	±0.08	±0.02	±0.24	±0.04	±0.14	±0.02	±0.07	±0.02	±0.16	±0.04

RH, relative humidity; MC, moisture content (%); IB, internal bond (N·mm^−2^).

## Data Availability

Data is contained within the article.
